# Antibacterial properties of marine algae incorporated polylactide acid membranes as an alternative to clinically applied different collagen membranes

**DOI:** 10.1007/s10856-024-06778-y

**Published:** 2024-01-29

**Authors:** Jan-Tobias Weitkamp, Soumaya El Hajjami, Yahya Acil, Johannes Spille, Selin Sayin, Emine Sükran Okudan, Eyüp Ilker Saygili, Salih Veziroglu, Christian Flörke, Peter Behrendt, Jörg Wiltfang, Oral Cenk Aktas, Aydin Gülses

**Affiliations:** 1 Department of Oral and Maxillofacial Surgery, University Hospital of Schleswig-Holstein, Campus Kiel, Arnold-Heller-Straße 3, 24105 Kiel, Germany; 2 Marine Science and Technology Faculty, Iskenderun Technical University, 31200 Iskenderun, Hatay Turkey; 3 Faculty of Fisheries, Akdeniz University, Dumlupınar Bulvarı, 07058 Antalya, Turkey; 4 Department of Medical Biochemistry, SANKO University, Sehitkamil, 27090 Gaziantep, Turkey; 5 Chair for Multicomponent Materials, Institute for Materials Science, Faculty of Engineering, Kiel University, Kaiserstr. 2, 24143 Kiel, Germany; 6 Department of Anatomy, Christian-Albrechts-Universität zu Kiel, Olshausenstr. 40, 24098 Kiel, Germany; 7 Department of Orthopedic and Trauma Surgery, Asklepios Skt. Georg, Hamburg, Germany

## Abstract

The reconstruction of bony defects in the alveolar crest poses challenges in dental practice. Guided tissue regeneration (GTR) and guided bone regeneration (GBR) procedures utilize barriers to promote bone regeneration and prevent epithelial growth. This study focuses on evaluating the antibacterial properties of marine algae-polylactic acid (PLA) composite membranes compared to commercially available collagen membranes. Marine algae (Corallina elongata, Galaxaura oblongata, Cystoseira compressa, Saragassum vulgare, and Stypopodium schimperi) were processed into powders and blended with PLA to fabricate composite membranes. Cytocompatibility assays using human periodontal ligament fibroblasts (*n* = 3) were performed to evaluate biocompatibility. Antibacterial effects were assessed through colony-forming units (CFU) and scanning electron microscopy (SEM) analysis of bacterial colonization on the membranes. The cytocompatibility assays demonstrated suitable biocompatibility of all marine algae-PLA composite membranes with human periodontal ligament fibroblasts. Antibacterial assessment revealed that Sargassum vulgare-PLA membranes exhibited the highest resistance to bacterial colonization, followed by Galaxaura oblongata-PLA and Cystoseira compressa-PLA membranes. SEM analysis confirmed these findings and revealed smooth surface textures for the marine algae-PLA membranes compared to the fibrous and porous structures of collagen membranes. Marine algae-PLA composite membranes show promising antibacterial properties and cytocompatibility for guided bone and tissue regeneration applications. Sargassum vulgare-PLA membranes demonstrated the highest resistance against bacterial colonization. These findings suggest that marine algae-PLA composite membranes could serve as effective biomaterials for infection control and tissue regeneration. Further in vivo validation and investigation of biodegradation properties are necessary to explore their clinical potential.

**Graphical Abstract:**

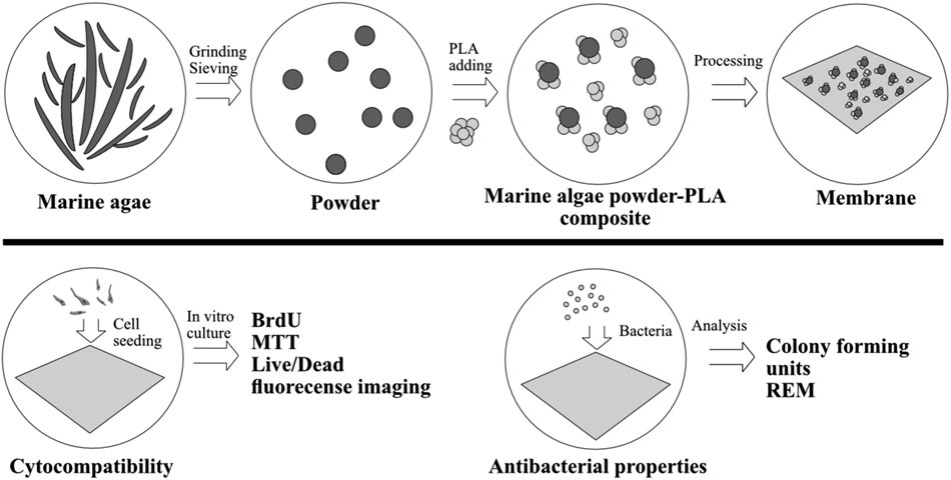

## Introduction

The reconstruction of the bony defects of the alveolar crest poses a great challenge for dental professionals. Guided tissue regeneration (GTR) and guided bone regeneration (GBR) procedures, where a barrier is utilized to allow bone regeneration and avoid fast proliferating epithelium growth on root and implant surfaces to provide space and time for slow-proliferating osteogenic (GBR) or periodontal ligament cells (GTR), has been nowadays a routine procedure in the daily dental practice [1].

The first clinically applied barrier membranes consisted of titanium meshes and expanded polytetrafluoroethylene (ePTFE) [2]. These non-resorbable barriers impose the risk of wound dehiscence secondary to challenging soft tissue closure, which subsequently leads to infections [3]. In addition, a second surgery is required for removal. Therefore, resorbable membranes derived from bioabsorbable materials, either of synthetic (aliphatic polyesters) or natural (collagen) origin, which are metabolized by hydrolysis or enzymatic activity respectively have been developed.

Collagen membranes are widely clinically applied in dentistry due to their proven biocompatibility and their promotion of wound healing. Besides autografts that only play an inferior role clinically, animal-derived bovine and porcine grafts are widely used in clinical praxis. These xenografts, however, impose risks like infection transmission and immogenicity [4]. In addition, due to religious constraints and a trend for vegan products, there is a need for cost-efficient natural bioresorbable alternatives.

**Fig. 1:**
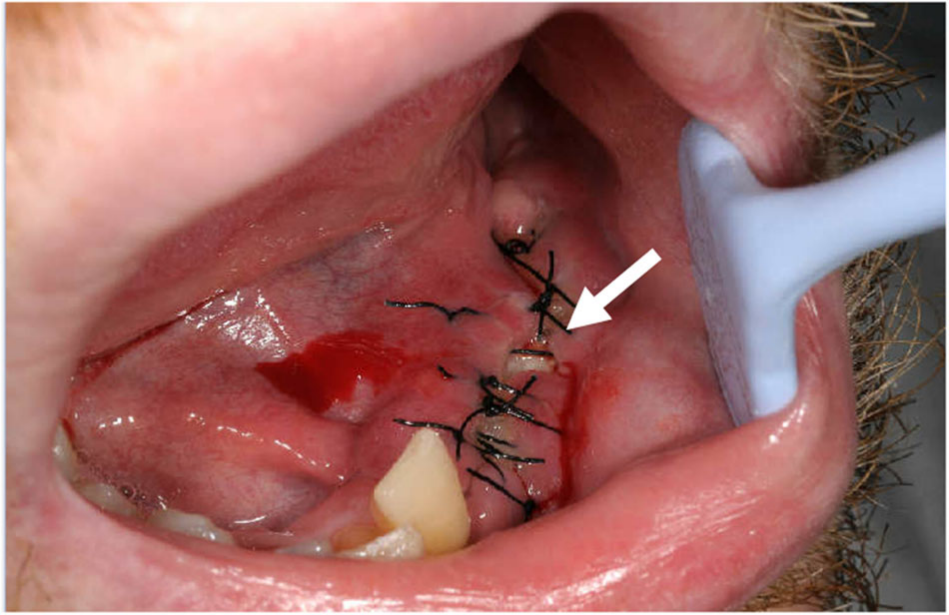
A clinical view of the membrane exposure (arrow) following guided bone regeneration

Infections secondary to GBR procedures could jeopardize the success of the procedure and require mostly the removal of biomaterials including the membrane and bone graft material used. Additionally, it has been reported that membrane exposure (Fig. 1), which leads to bacterial contamination has a significant influence on the outcome of bone augmentation. Garcia et al. [5] have reported that the sites without membrane exposure achieved 74% more horizontal bone gain than the sites with exposure. Prado-Prone et al. have also suggested that the outcome of the GBR procedure is commonly affected by the local bacterial colonization of the membrane causing postoperative infections and a premature rupture of the membrane limiting the regeneration process [6]. Therefore, resistance against bacterial contamination could improve the success rate of GBR/GTR procedures.

A potential source for tissue engineering applications and resorbable membranes are marine-based natural biopolymers due to their biocompatibility, biodegradability and their natural abundance [7]. They have been introduced in numerus fields in regenerative medicine. In this regard, marine algae are of great interest as a source of natural biopolymers due to their abundance and inexpensive extraction [8]. In the past years, a wide spectrum of bioactivities such as antitumoral, antiviral, antioxidant and cell proliferative effects have been observed which makes them ideal biomaterials for medical applications [9, 10]. After being processed into powders they can be blended with polyesters to enhance their biomechanical properties and thereby increase their structural integrity which enables medical applications like membranes for periodontal and implantology surgery [11]. In this regard, poly(caprolactone) (PCL), poly (hydroxy alkanoates) (PHA), and polylactide (PLA) have been utilized. In recent studies, a manufacturing process for marine algae by using the extracts of red and brown algae *Corallina elongata*, *Galaxaura oblongata*, *Cystoseira compressa*, *Saragassum vulgare* and *Stypopodium schimperi* has been established as de novo alternatives to porcine-derived collagen membranes.

It has been well known that algae have evolved to survive the high concentrations of infectious and surface-fouling bacteria that are indigenous to ocean waters and contain pharmacologically active compounds such as phlorotannins, fatty acids, polysaccharides, peptides, and terpenes which combat bacterial invasion [12]. Despite promising results regarding their biocompatibility as in PLA embedded membranes, there is still an ongoing need for the assessment of the antibacterial properties of these materials. The aim of the present study was to identify antibacterial effects of composite membranes compared to clinically applied porcine and bovine membranes. Therefore, PLA composites were fabricated using *Corallina elongata*, *Galaxaura oblongata*, *Cystoseira compressa*, *Saragassumi vulgare* and *Stypopodium schimperi* and investigated with respect to cytocompatibility and bacterial colonization.

## Material and methods

All experiments were performed after receiving approval from The Ethics Committee of the Medical Faculty of Christian Albrechts University, Kiel, Germany. (D640/20) and was conducted according to the guidelines of the Helsinki Declaration of Human Rights. Human fibroblasts were obtained from patients who had undergone oral surgical interventions such as tooth extraction third molar removal, etc. at the Oral and Maxillofacial Surgery Department at the Christian Albrechts University Hospital Schleswig-Holstein, Kiel Campus, Kiel, Germany.

### Fabrication of marine algae-PLA composites

Marine algae-PLA composites were manufactured as previously described [7, 8]. Briefly, powder of five different types of algae; Corallina elongata, Galaxaura oblongata, Cystoseria compressa, Sargassum vulgare, and Stypopodium schimperi were collected at the Mediterranean Sea. After identification, water soluble components were removed, the materials were grinded and then vacuum dried at 50 °C for 24 h. Micro scale marine-algae powder (MAP) were washed with acetone and dried in an oven at 80 °C for 24 h. Then PLA (Goodfellow, 459-898- 81) and MAP were mixed in a mechanical mixer operating at 150 °C for 15 min at a rotor speed of 50 rpm. The algae powder made up 5–10% of the total mixture. After mixing, the composites were pressed into thin plates with a custom hot press at 180 °C and 8 MPa for 20 min and then placed in an oven for cooling Afterwards, thin plates were cut into 1 cm^2^ sample dimensions for further characterization. These samples were named as: Type I MAP-PLA (Corallina elongata), Type II MAP-PLA (Galaxaura oblongata), Type III MAP-PLA (Cystoseria compressa), Type IV MAP-PLA (Sargassum vulgare) and Type V MAP-PLA (Stypopodium schimperi).

### Cytocompatibility of marine algae-PLA membranes

Human fibroblasts were isolated from gingival tissues from the patients who underwent surgeries in the Department of Oral and Maxillofacial Surgery at Christian-Albrechts-University in Kiel, Germany. (The protocol was conducted according to the guidelines of the Declaration of Helsinki and approved by the Institutional Ethics Committee of the Medical Faculty of Christian Albrechts University, Kiel-Germany /protocol code D640/20.) Briefly, adherent gingival tissues were cut into fragments of 0.3–0.5 cm in diameter, and extensive washing in phosphate-buffered solution (pH:7.4) was performed. The fragments were seeded as explants into the culture flasks and cultivated at 37 °C in a humidified atmosphere of 95% air and 5% CO_2_. Dulbecco’s modified eagle’s medium, supplemented with fetal calf serum, 100,000 mg L^−1^ penicillin, 100 mg L^−1^ streptomycin, 2 mM l-glutamine, 100 nM dexamethasone and 1 mM l-ascorbic acid 2-phosphatase (Sigma, Deisenhofen, Germany) was used for cultivation. Cells were subcultures in a second passage at a density of 3.3 × 10^6^ cm^−2^. For the second passage, a cell dispenser was used to bring the cells into suspension. One hundred microliters containing 3.3 × 10^6^ fibroblast-like cells in the second passage were transferred onto the algae-PLA membranes. As a positive control clinically applied bovine (Mem-Lok®) and porcine (Argonaut®) resorbable collagen membranes (RCM; both Biohorizon, Birmingham, USA) were used. Internal controls were human PDL fibroblasts in monolayers. After incubation for 24 h in culture ((89% Dulbecco’s modified Eagle´s minimum essential medium- PAA Laboratories, Austria, 10% fetal calf serum (FCS; Biochrom, Germany, 1% Penicillin/Streptomycin, Biochrom, Germany). medium at 37 °C in an atmosphere of 5% CO_2_, methyltransferase assay (MTT, In vitro Cell Proliferation KIT 1; Roche, Mannheim, Germany) and 5-bromo-2’-deoxyuridine BrdU (Cell Proliferation ELISA; Roche Diagnostics, Mannheim, Germany) assay were performed according to manufacturer’s instructions to quantitatively analyze cytocompatibility. Briefly, after cultivation of fibroblasts for 24 h in a 96-well plate, 10 µl of MTT labeling reagent was added to each well. The samples were incubated for 4 h at an atmosphere of 37 °C with 5% CO_2_. Metabolic cell activity was then detected by a Multi-scan MS spectrophotometer (Labsystems, Stockholm, Sweden) at 550 nm absorbance. For BrdU assay, human fibroblasts were cultured in 100 µl culture medium for 24 h. Afterwards, 10 µl BrdU labeling reagent was added per well. The cells were incubated for 2 h at 37 °C and then the supernatant was removed. The following steps were performed accorting to manufacurer’s instruction. Finally, the optical density of the individual samples was measured using a microplate reader (Spectra Max plus 384, Molecular Devices, Sunnyvale, CA, USA) photometrically at 450 nm wavelength.

### Live/Dead immunostaining and fluorescence imaging

In addition to cytocompatibility assays (see above) cell viability was identified by Live/Dead staining using fluorescein diacetate (FDA; Sigma-Aldrich, MO, USA) and propidium iodide (PI, Sigma-Aldrich, St. Louis, MO, USA). Human PDL fibroblasts were seeded at a density of 10^4^ cells/MAP-PLA and porcine and bovine membrane. After incubation in culture medium for 15 min in dark eviroment, samples were washed with PBS and subsequently stained with the FDA solution containing 30 µL of stock solution (1 mg FDA/mL acetone) diluted in 10 mL PBS. After an incubation period of 15 min at a humified atmosphere of 37 °C in a dark environment, the FDA solution was removed and replaced by 500 µL PI stock solution. After an incubation time of 120 s, the cells were then washed twice with PBS. Afterwards, the live and dead cells were detected by fluorescence imaging using a fluorescence microscope (Axioplan2). Representative images of MAP-PLA composites and commercial membranes were taken with detection of green fluorescence (FDA) at 530 nm and red fluorescence (PI) at 620 nm.

### Colony-forming units

To quantify bacterial colonization of algae-PLA composites and porcine and bovine collagen membranes, colony-forming units (CFU) were used to estimate the number of viable bacteria. A cultivation with 10 ml sterile nutrient solution (BHI, Brain–Heart-Infusion Broth, Carl Roth GmbH + Co. KG, Karlsruhe, Germany) and 100 μl bacterial culture with E. faecalis (ATCC 29,212) was conducted at 37 °C for 24 h. (Heraeus B6060, Heraeus Holding GmbH, Hanau, Germany). Afterwards, the boxes (Eppendorf pipette tip reusable boxes Eppendorf AG, Hamburg, Germany) containing the models were sterilized at 121 °C (Autoclave Melag Vacuklav 24, MELAG Medizintechnik oHG, Berlin, Germany). On the first day, the membranes were infected with 200 ml of sterile BHI and 100 μl of the overnight culture and then incubated at 37 °C (Scientific C24 Incubator Shaker, New Brunswick Scientific, Edison, New Jersey, USA). After 4 h, the optical density was controlled via spectrophotometer (BioPhotometer 6131, Eppendorf AG, Hamburg, Germany) at 600 nm (OD600), which was set to 0.8. Nutrient solution was exchanged in every 24 h with 200 ml of sterile BHI for 6 days. All experiments were conducted in triplicate.

### Scanning electron microscopy

Bacterial colonization on MAP-PLA and commercial membranes was besides CFU semi quantitatively evaluated using scanning electron microscopy (SEM). After incubation in a humified atmosphere of 37 °C at 5% CO_2_, the culture medium was removed and cells were fixed with glutaraldehyde (Sigma, St. Louis, USA) 3% in PBS at a pH value of 7.4 for 24 h. After removal of the glutaraldehyde solution, the samples were dehydrated in an ascending alcohol dilution for 300 s for each series. After that, drying with hexamethyldisilane for 1 min (Sigma-Aldrich, St. Louis, USA) and a gold vapor deposition with a thickness of 15 nm (SCD 500, CAL-Tec, Ashford, UK) were performed and SEM analysis (Jeol, Freising, Germany) was conducted at a voltage between 10–15 kV.

### Statistics

All experiments were conducted in triplicate. All data were tested for normality using the Kolmogorov– Smirnov test. Statistical analysis was performed using Graph Pad prism 5 program (San Diego, CA, USA). Oneway analysis of variance (ANOVA) with Bonferroni’s multiple comparisons was used to compare means among the independent experimental groups. Differences were considered significant if *P* ≤ 0.05. Quantitative data in the text are presented as mean and standard deviation (SD).

## Results

### Cytocompatibility of MAP-PLA and RCM

**Fig. 2:**
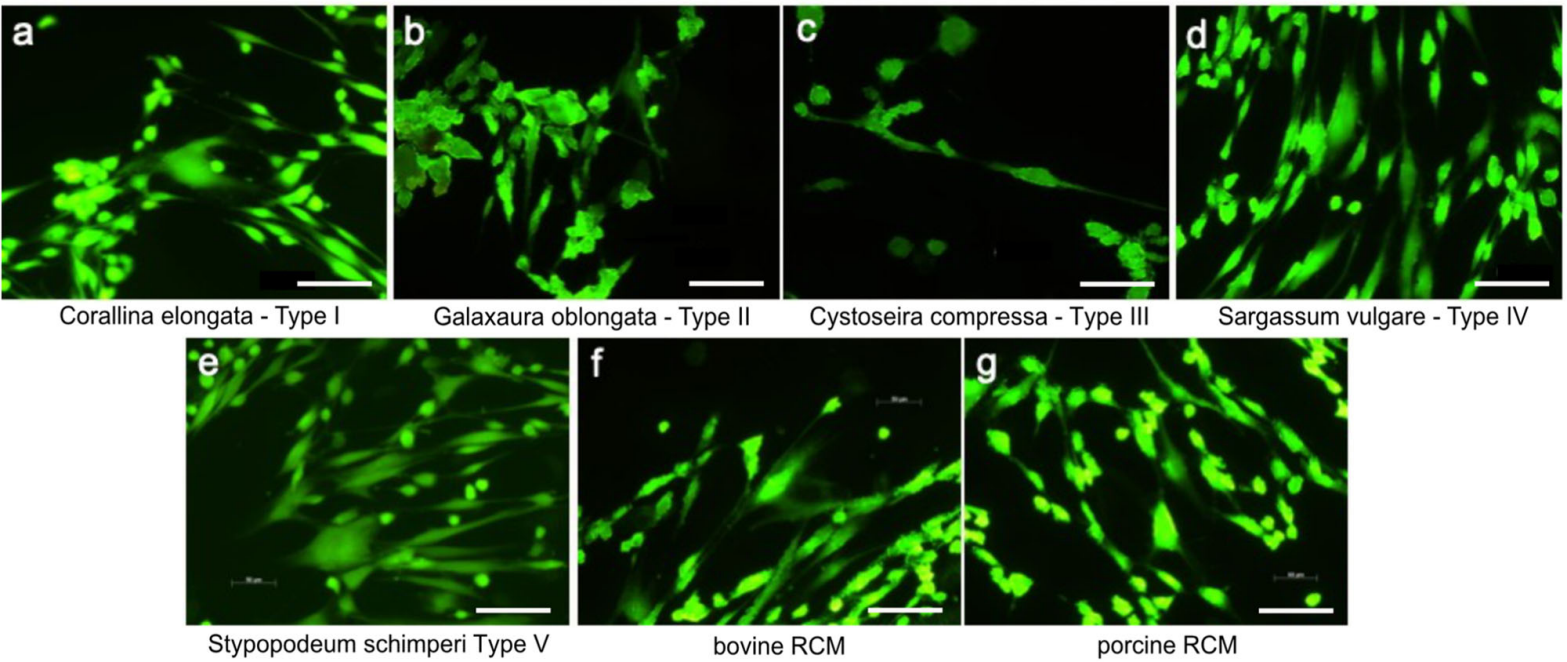
**Cell viability with fluorescence imaging**. Representative images of L/D immunostaining of fibroblasts seeded on MAP-PLA membranes (**a**–**e**) and bovine and porcine RCM (**f** + **g**). Bar 10 µm. Fluorescence imaging revealed viable cells on type I-V grafts as well as the clinically applied products. MAP-PLA composites manufactured with *Cystoseira compressa* appeared slightly less colonized compared to all other experimental groups (**c**)

Cytocompatibility of MAP-PLA and RCM was evaluated by BrdU and MTT assay as well as L/D immunostaining. BrdU assay revealed no significant differences among type I-V MAP-PLA and RCM groups. Metabolic cell activity in MTT assay was significantly lower on *Cystoseira compressa* and *Stypopodeum schimperi* compared to bovine and porcine RCM (type IV vs bovine RCM: *p* < 0.0001; type IV vs porcine RCM: *p* = 0.0195; type V vs borth RCM: *p* < 0.0001). In general, all experimental groups resulted in high cell viability and metabolic activity. This was in accordance with L/D staining of MAP-PLA and RCM seeded biomaterials (Fig. 2a–g).

**Fig. 3:**
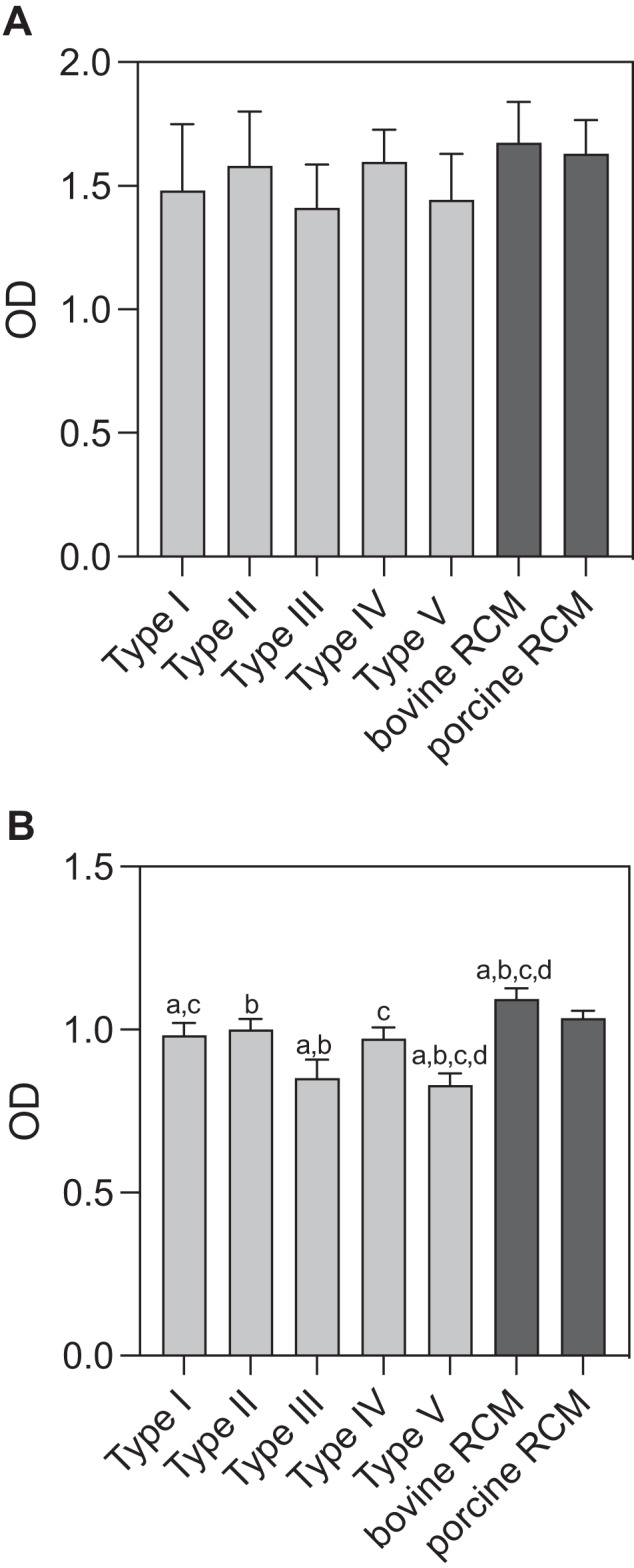
**Cytocompatibility of MAP-PLA membranes and RCMs. A** BrdU and **B** MTT assay of fibroblasts seeded on MAP-PLA type I-V (Corallina elongata, Galaxaura oblongata, Cystoseira compressa, Saragassum vulgare and Stypopodium schimperi) and bovine and porcine RCM after 24 h. OD = optical density. a-d groups that are statistically significant to one another. Oneway-Anova, *p* < 0.05

In summary, all MAP-PLA membranes revealed sufficient cytocompatibility of human PDL fibroblasts compared to clinically applied bovine and porcine RCM. Fibroblasts seeded on Stypopodium schimperi-PLA membranes had the lowest metabolic cell activity after 24 h. (Fig. 3)

### Bacterial colonization of MAP-PLA and RCM

**Fig. 4:**
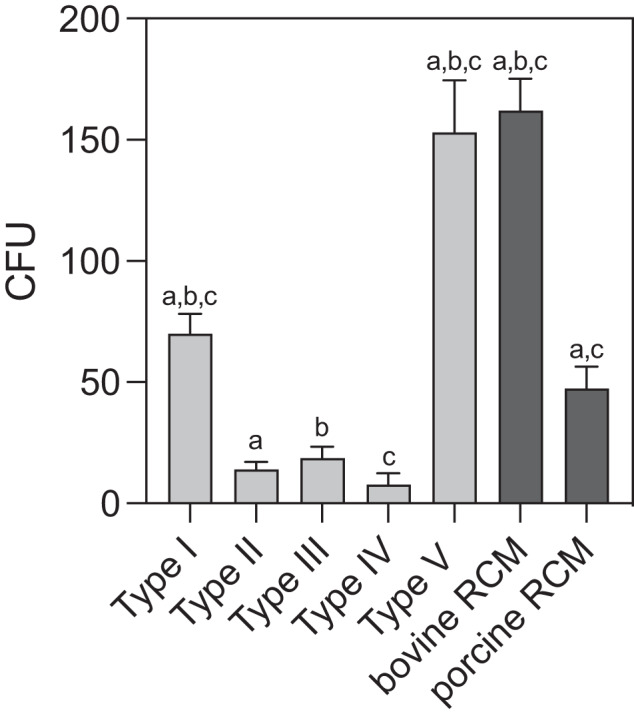
**CFU of entereococcus faecalis on MAP-PLA and RCM**. Bacterial colonization after 5 days of incubation. MAP-PLA type I-V are Corallina elongata, Galaxaura oblongata, Cystoseira compressa, Saragassum vulgare and Stypopodium schimperi. a–d groups that are statistically significant to one another. Oneway-Anova, *p* < 0.05

Antibacterial properties of MAP-PLA composites and RCMs were detected by CFU of enterococcus faecalis and SEM analysis of bacterial seeded membranes. Quantitative analysis of CFU revealed significant differences between RCM and MAP-PLA groups as well as among algae membranes itself. Overall type II, III and IV showed the fewest bacterial colonization. Saragassum vulgare membranes displayed 7.76 ± 4.73 CFU which was the lowest amount detected among all experimental groups. This result differed significantly from both RCMs (*p* < 0.001) and additionally from Corallina elongata-PLA (*p* = 0.0001) and Stypopodeum schimperi (*p* < 0.0001). Besides type IV MAP-PLA membrane type II and III showed only little enterococcus faecalis growth (type II 14 ± 3 CFU, type III 18.67 ± 4.73 CFU. Corallina elongata-PLA membranes demonstrated more bacterial growths compared to the porcine RCM and Stypopodeum schimperi-PLA membrane resulted in the highest bacterial colonized surface with 153 ± 21.63 CFU (Fig. 4 and Table 1).

**Table 1:** Descriptive results of CFU of enterococcus faecalis on MAP-PLA and RCM

	CFU on MAP-PLA and RCM			
Membrane type	M [CFU/100 µl]	Md [CFU/100 µl]	Min [CFU/100 µl]	Max [CFU/100 µl]
Corallina elongate Type I	2.8 × 10^6^	2.7 × 10^6^	2.5 × 10^6^	3.2 × 10^6^
Glaxaura oblongata Type II	5.6 × 10^6^	5.6 × 10^6^	4.4 × 10^6^	6.8 × 10^6^
Cystoseira compressa Type III	7.5 × 10^6^	6.8 × 10^6^	6 × 10^6^	9.6 × 10^6^
Saragassum vulgare Type IV	0.031 × 10^6^	0.024 × 10^6^	0.016 × 10^6^	0.052 × 10^6^
Stypopodium schimperi Type V	61.2 × 10^6^	54 × 10^6^	54 × 10^6^	70.8 × 10^6^
Bovine RCM	64.8 × 10^6^	62.8 × 10^6^	60.8 × 10^6^	70.8 × 10^6^
Porcine RCM	18.9 × 10^6^	19.2 × 10^6^	15.2 × 10^6^	22.4 × 10^6^

**Fig. 5:**
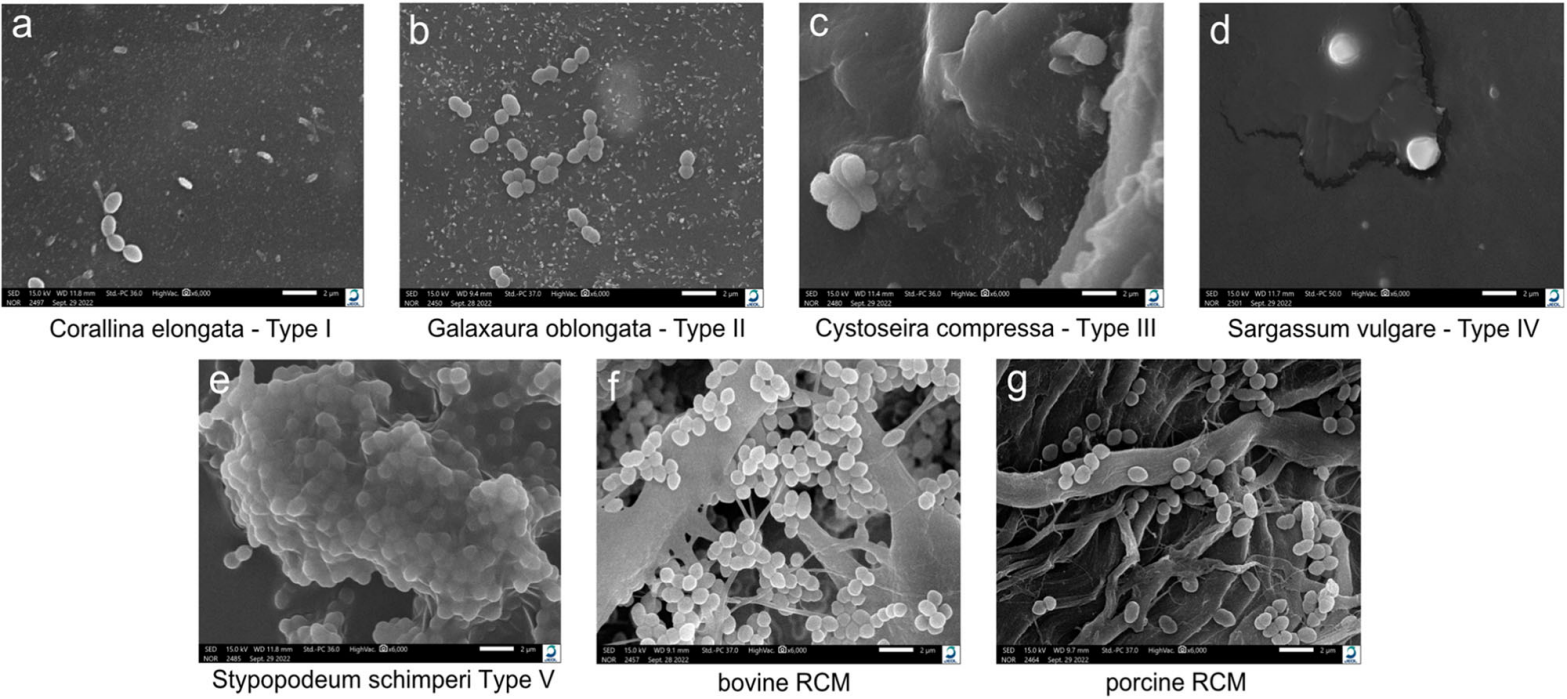
**Scanning electron microscopy of enterococcus faecalis on MAP-PLA and RCMs**. Representative SEM images of bacterial colonization and surface textures of MAP-PLA membranes (**a**–**e**) and bovine and porcine RCM (**f** + **g**). Bar 2 µm

SEM results confirmed the findings obtained by CFU. As shown in Fig. 5, type V, bovine RCM and porcine RCM show highly adherent enterococcus faecalis colonies (Fig. 5e–g) respectively. Furthermore, there are distinct differences in membrane surface texture. Compared to RCM membranes the surface areas of the MAP-PLA membranes appear homogenous and smooth. The bovine and porcine RCM revealed a fibrous texture with porous structures. Bacterial colonization can be seen in deeper layers throughout the pores of the membrane (Fig. 5f–g). In summary, Sargassum vulgare-PLA membranes revealed minimal bacterial adhesion and growth characteristics.

## Discussion

Infection control is a major determinant of guided tissue regeneration. The aim of this study was to investigate antibacterial properties of different marine algae-PLA membranes compared to commercially available resorbable collagen ones used for guided bone/tissue regeneration. Despite its limitations such as mono-bacterial colonization model -which differs from the oral microbiota- and ex-vivo study design, current study has clearly showed that the Sargassum vulgare embedded membranes followed by Galaxaura oblongata and Cystoseira compressa showed superior resistance against bacterial colonization compared to all other subgroups. The bacterial colonization on Corallina elongata and Stypopodium schimperi, which presented similar characteristics compared to the collagen subgroups showed that, despite their identical classification, the algae could have different biological effects. Therefore, the selection of an algae for a possible clinical application should be performed with consideration of its biological characteristics and the clinical scenario. Together with their sufficient cytocompatibility the brown algae facilitate great potential for biomedical applications.

All marine algae -PLA composite membranes experimented herein showed suitable cytocompatibility as BrdU and MTT assays revealed. The results were equal to those of clinically applied RCMs, which confirms the results of the previous studies of our research group [9, 10]. In general, it is known that various types of marine algae are cytocompatible polymers that can be used for biomaterial development. In addition, all marine algae displayed a homogenous surface colonization of fibroblasts as fluorescence imaging revealed. Therefore, it can be assumed that the membranes are well-suited for in-vivo applications.

Interestingly, marine algae -PLA composite membranes showed antibacterial properties as CFU of Enterococcus faecalis and SEM revealed. In detail, Type II (Galaxaura oblangata), Typ III (Cystoseira compressa) and Typ IV (Sargassum vulgare) composites demonstrated sufficient antibacterial properties in contrast to membranes that were fabricated using Corallina elongata and Stypopodium schimperi powder. Sargassum vulgare showed superior results compared to other marine algae types and especially when compared to RCMs. It is known that solvent extracts from Sargassum vulgare have antibacterial activity for B. subtilis, S. aureus, E. coli, S. typhi, K. pneumoniae, C. albicans [13]. The results of the present study suggest that it is also effective against E. faecalis. It is very well known that the bacterial colorizations of the oral aperture are generally multi-bacterial and E. faecalis is usually not a dominant species in these infections. However, several studies have showed that E. faecalis appears in many apical infections and may vegetate in bone after extraction of an infected tooth and colonize a dental implant after placement in the healed site, which may cause an implant failure secondary to marginal bone loss [14]. Due to the risk of an infection, the exposition of the membrane could jeopardize the bone/tissue regeneration and necessitates often the removal of the biomaterials used. The confirmation of the current results via an in-vivo multi-bacterial colonization model might be beneficial in developing novel membranes showing antibacterial resistance without impairing the cytocompatibility.

To control membrane-associated infections, a loading with antibiotics has been introduced in preclinical studies [15]. Parallel to the advancements in the production of nanofibers, antibiotic-loaded electrospun-nanofibers have become the main focus of various studies to develop a novel antibacterial strategy in guided bone/tissue regeneration [16, 17]. In a recent article, Ing-Hua et al. [18] have used Poly(D,L-lactic acid) nanofibers encapsulated amoxicillin and showed that inhibited bacterial growth and promoted the viability and mobility of periodontal ligament cells. Despite its limitations, such as the use of an ex-vivo experimental model with mono-bacterial decontamination, the promising results of the current study could be a focus of a future study comparing the antibacterial effects of marine algae-PLA with antibiotic-loaded nanofiber structures. However, the technical requirements for the production of nanofibers are significantly higher compared to the marine algae-PLA membranes, thus electrospun fibers are fabricated by dissolving a polymer in an organic solvent and using a high force electric field to draw nano- to micro-scale fibers [19]. Besides that, it should be kept in mind that the antimicrobial agents used in the production of antibiotic-doped-nanofibers could induce allergic reactions [20].

The antibacterial properties of marine algae are based on natural compounds that can be synthetized by see weeds like polysaccharides, fatty acids, phlorotannins, pigments, lectins, alkaloids, terpenoids and halogenated compounds. They are part of the natural defense against bacterial infestation in their habitat. In addition to biomaterial development, marine algae compounds are also under investigation for unconventional drugs that can target new diseases or multi-resistant strains of pathogenic microorganisms. A possible way to overcome antibiotic resistance.

It is well known that RCM membranes have no antibacterial properties [21]. They are much more likely to provide a suitable ground for bacterial growth and wound infections. In addition to the incorporated antibacterial properties, the nature of the surface of the marine algae-PLA membranes also seems to play a decisive role in bacterial colonization. The SEM images showed a significantly smoother and more homogeneous surface for the marine algae-PLA membranes compared to the RCMs. Bacterial colonization was observed through all depts of the membrane. In the literature, it is stated that commercially RCM as well as ePTFE membranes have only little barrier effects against bacteria. They can pass through these membranes leading to deeper wound infections and impairing cell attachment like periodontal ligament fibroblasts [22].

## Conclusion

Membranes of Sargassum vulgare-PLA composites exhibit promising results in guided tissue regeneration. Further in-vivo validation via multi-bacterial experimental models and assessment of biodegradation properties is now needed to take another step towards clinical application.

## Data Availability

The datasets used and/or analysed during the current study available from the corresponding author (jan-tobias.weitkamp@uksh.de) on reasonable request.
